# Fast protein structure searching using structure graph embeddings

**DOI:** 10.1093/bioadv/vbaf042

**Published:** 2024-03-05

**Authors:** Joe G Greener, Kiarash Jamali

**Affiliations:** Medical Research Council Laboratory of Molecular Biology, Cambridge, CB2 0QH, United Kingdom; Medical Research Council Laboratory of Molecular Biology, Cambridge, CB2 0QH, United Kingdom

## Abstract

Comparing and searching protein structures independent of primary sequence has proved useful for remote homology detection, function annotation, and protein classification. Fast and accurate methods to search with structures will be essential to make use of the vast databases that have recently become available, in the same way that fast protein sequence searching underpins much of bioinformatics. We train a simple graph neural network using supervised contrastive learning to learn a low-dimensional embedding of protein domains.

**Availability and implementation:**

The method, called Progres, is available as software at https://github.com/greener-group/progres and as a web server at https://progres.mrc-lmb.cam.ac.uk. It has accuracy comparable to the best current methods and can search the AlphaFold database TED domains in a 10th of a second per query on CPU.

## 1 Introduction

A variety of methods have been developed to compare, align, and search with protein structures (Hasegawa and Holm 2009). Since structure is more conserved than sequence (Illergård *et al.* 2009) these methods have proved useful in remote homology detection (Al-Fatlawi *et al.* 2023), protein classification (Sillitoe *et al.* 2021), inferring function from structure (Zhang *et al.* 2017), clustering large databases (Barrio-Hernandez *et al.* 2023, Durairaj *et al.* 2023), and assessing the accuracy of structure predictions. Global coordinate comparisons like TM-align (Zhang and Skolnick 2005) provide interpretable scores that are comparable across protein size, with a challenge being how to align the residues independent of the primary sequence. Mathematical representations of 3D space such as 3D Zernike descriptors (Guzenko *et al.* 2020, Aderinwale *et al.* 2022) avoid this issue but are limited in accuracy. Other approaches include comparing residue–residue distances (Taylor and Orengo 1989, Liu *et al.* 2018, Holm 2020, Guo *et al.* 2024), which can access precise geometries conserved in the structural core, and considering local geometry (Shindyalov and Bourne 1998). The highest accuracy methods tend to be careful comparisons based on coordinates like Dali (Holm 2020), but searching large structural databases such as the AlphaFold Protein Structure Database (Jumper *et al.* 2021, Varadi *et al.* 2022) or the ESM Metagenomic Atlas (Lin *et al.* 2023) with these methods is slow. Recently, Foldseek (van Kempen *et al.* 2024) has addressed this problem by converting protein structure into a sequence of learned local tertiary motifs. It then uses the rich history of fast sequence searching in bioinformatics to dramatically reduce the pairwise comparison time of the query with each member of the database. It follows that to further reduce search time, the pairwise comparison step should be made even faster.

Inspired by the impressive performance of simple graph neural networks (GNNs) using coordinate information for a variety of molecular tasks (Satorras *et al.* 2021), we decided to train a model to embed protein domains into a low-dimensional representation. Two embeddings can be compared very quickly by cosine similarity and a query can be compared to each member of a pre-embedded database in a vectorized manner on CPU or GPU. It makes sense to use expertly-curated classifications of protein structures when training such an embedding (Cheng *et al.* 2014, Chandonia *et al.* 2019, Sillitoe *et al.* 2021); we use supervised contrastive learning (Khosla *et al.* 2020) to allow the embedding to be learned in a manner that reflects such an understanding of protein structure space and returns search results consistent with it.

A number of recent methods have used protein structure graph embeddings (Chen *et al.* 2021, Xia *et al.* 2022, Kandathil *et al.* 2024) and contrastive learning (Xia *et al.* 2022, Hamamsy *et al.* 2023a, Luo and Luo 2023). Embedding protein folds has also been done using residue-level features (Villegas-Morcillo *et al.* 2021, Durairaj *et al.* 2023), and GNNs acting on protein structure have been used for function prediction (Gligorijević *et al.* 2021). Other studies have used unsupervised contrastive learning on protein structures and show that the representations are useful for downstream prediction tasks including protein structural similarity (Chen *et al.* 2023, Hermosilla and Ropinski 2022, Zhang *et al.* 2022). Contrastive learning using protein classifications has also improved language models for protein sequences, showing clustering that better preserves protein structure space (Heinzinger *et al.* 2022). Protein structure has been incorporated into language models more broadly, often with the intention of searching for remote homology (Hamamsy *et al.* 2023b, Liu and Shen 2023, Zheng *et al.* 2023, Heinzinger *et al.* 2024, Iovino *et al.* 2024, Liu *et al.* 2024). Progres provides a fast and accurate alternative to these methods, with the ability to search the AlphaFold TED domains (Lau *et al.* 2024), that is available as a web server and as software.

## 2 Methods

### 2.1 Training

Structures in the Astral 2.08 95% sequence identity set including discontinuous domains were used for training (Chandonia *et al.* 2004). We chose 400 domains randomly from the Astral 2.08 40% sequence identity set to use as a test set (see below) and another 200 domains to use as a validation set to monitor training. We removed domains with 30% or greater sequence identity to these 600 domains using MMseqs2 (Steinegger and Söding 2017), and also removed domains with fewer than 20 or more than 500 residues. This left 30 549 domains in 4862 families for training.

mmCIF files were downloaded and processed with Biopython (Cock *et al.* 2009). Some processing was also carried out with BioStructures.jl (Greener *et al.* 2020). Cα atoms were extracted for the residues corresponding to the domain. Each Cα atom is treated as a node with the following features: number of Cα atoms within 10 Å divided by the largest such number in the protein, whether the Cα atom is at the N-terminus, whether the Cα atom is at the C-terminus, the *τ* torsion angle between Cα_*i*__−1_/Cα/Cα_*i*__+1_/Cα_*i*__+2_, and a 64D sinusoidal positional encoding for the residue number in the domain (Vaswani *et al.* 2017).

PyTorch was used for training (Paszke *et al.* 2019). The neural network architecture was similar to the *E*(*n*)-equivariant GNN in Satorras *et al.* (2021). We used a PyTorch implementation (https://github.com/lucidrains/egnn-pytorch) and a configuration similar to the molecular data prediction task, i.e. not updating the particle position. In this case, the model is analogous to a standard GNN with relative squared norms inputted to the edge operation (Satorras *et al.* 2021). Edges are sparse and are between Cα atoms within 10 Å of each other. Six such layers with residual connections are preceded by a one-layer multilayer perceptron (MLP) acting on node features and followed by a two-layer MLP acting on node features. Node features are then sum-pooled and a two-layer MLP generates the output embedding, which is normalized. Each hidden layer has 128 dimensions and uses the Swish/SiLU activation function (Hendrycks and Gimpel 2016), apart from the edge MLP in the GNN which has a hidden layer with 256 dimensions and 64D output. The final embedding has 128 dimensions.

Supervised contrastive learning (Khosla *et al.* 2020) is used for training. Each epoch cycles over the 4862 training families. For each family, five other families are chosen randomly. For each of these six families, six domains from the family present in the training set are chosen randomly. If there are fewer than six domains in the family, duplicates are added to give six. This set of 36 domains with six unique labels is embedded with the model and the embeddings are used to calculate the supervised contrastive loss with a temperature of 0.1 (Khosla *et al.* 2020). During training only, Gaussian noise with variance 1.0 Å is added to the *x*, *y*, and *z* coordinates of each Cα atom. Training was carried out with the Adam optimizer (Kingma and Ba 2014) with learning rate $5\times 10^{-5}$ and weight decay $1\times 10^{-16}$. Each set of 36 domains was treated as one batch. Training was stopped after 500 epochs and the epoch with the best family sensitivity on the validation set was used as the final model. Training took around a week on one RTX A6000 GPU.

### 2.2 Testing

For testing, a similar approach to Foldseek was adopted (van Kempen *et al.* 2024). The 15 177 Astral 2.08 40% sequence identity set domains were embedded with the model. The embeddings are stored as Float16 to reduce the size of large databases on disk, but this has no effect on search performance as shown in Supplementary Table S1. Four hundred of these domains were chosen randomly and held out of the training data as described previously. Like Foldseek, we only chose domains with at least one other family, superfamily, and fold member. For each of these 400 domains, the cosine similarity of embeddings to each of the 15 177 domains was calculated and the domains ranked by similarity with the query domain included. For each domain, we measured the fraction of TPs detected up to the first incorrect fold detected. TPs are same family in the case of family-level recognition, same superfamily, and not same family in the case of superfamily-level recognition, and same fold and not same superfamily in the case of fold-level recognition. We also report the mean TM-align score and the fraction of hits with the same fold for the top 20 hits for each query.

All CPU methods were run on an Intel i9-10980XE CPU and with 256 GB RAM. Progres, Foldseek, and MMseqs2 were run on 16 threads. The GPU methods were run on a RTX A6000 GPU. Progres was run with PyTorch 1.11. Foldseek version 8.ef4e960 was used. For TM-align, we used the fast mode, which has similar performance to the normal mode (van Kempen *et al.* 2024). For 3D-SURFER, the neural network model and mainchain atoms were used. EAT was run with the “–use_tucker 1” flag. ESM-2 embeddings used the esm2_t36_3B_UR50D model which has a 2560D embedding. The mean of the per-residue representations was normalized and comparison between sequences was carried out with cosine similarity. For MMseqs2, easy-search with a sensitivity of 7.5 was used.

For contact order, all residue pairs with Cβ atoms (Cα for glycine) within 8 Å are considered. The contact order of a structure is then defined as


$$\frac{\sum_{i}^{N}S_{i}}{LN}$$

where $S_{i}$ is the sequence separation of the residues in contacting pair i, N is the number of contacting pairs, and L is the sequence length of the protein.

### 2.3 Databases

The AlphaFold database domain embeddings were prepared from the TED set of domains (Lau *et al.* 2024) using cluster representatives from clustering at 50% sequence identity. Clustering was carried out with MMseqs2 using the command “mmseqs easy-cluster ted_100.fasta clusterRes tmp –min-seq-id 0.5 -c 0.9 –cov-mode 5 -s 7.5”. This gave 53 344 209 clusters, fewer than the TED analysis due to the use of easy-cluster over easy-linclust. The FAISS (Douze *et al.* 2024) index was prepared using “IndexFlatIP(128)”, which carries out exhaustive searching using the same cosine similarity as Progres. Query structures may be automatically split into domains before searching using Chainsaw (Wells *et al.* 2024), with each domain searched separately.

### 2.4 Web server

The web server was implemented in Django and uses 3Dmol.js (Rego and Koes 2015) for visualization.

## 3 Results

**Figure 1.**
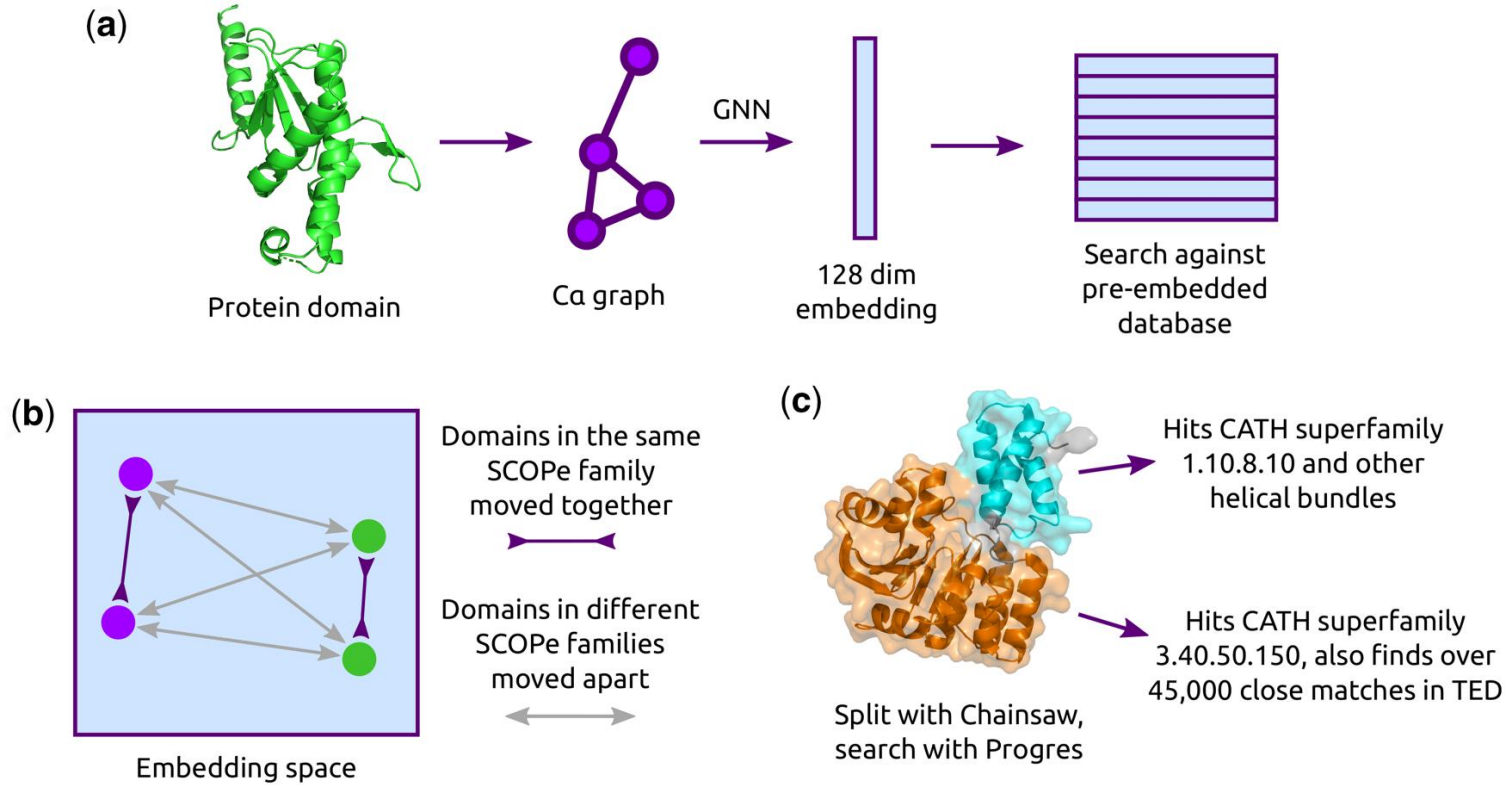
Protein structure embedding. (a) Protein domains are treated as a graph with Cα atoms as nodes and edges between Cα atoms within 10 Å. A GNN embeds the graph into a 128D representation. This can be compared quickly to a pre-embedded search database. (b) Supervised contrastive learning (Khosla *et al.* 2020) is used to train the model, with embeddings for domains in the same SCOPe family pushed together and embeddings for domains in different SCOPe families pushed apart. Structures can be automatically split into domains before searching. (c) Running Progres on a bacterial methyltransferase (PDB ID 3FRH) (Schmitt *et al.* 2009).

We trained a simple GNN, called Progres (PROtein GRaph Embedding Search), to embed a protein structure independent of its sequence (see Fig. 1a). Since we use distance and torsion angle features based on coordinates the embedding is SE(3)-invariant, i.e. it does not change with translation or rotation of the input structure. As shown in Fig. 1b, supervised contrastive learning (Khosla *et al.* 2020) on SCOPe domains (Chandonia *et al.* 2019, Andreeva *et al.* 2020) is used to train the model, moving domains closer or further apart in the embedding space depending on whether they are in the same SCOPe family or not. Sinusoidal position encoding (Vaswani *et al.* 2017) is also used to allow the model to effectively use information on the sequence separation of residues. The main intended use of such an embedding is fast searching for similar structures by comparing the embedding of a query structure to the pre-computed embeddings of a database of structures. Our model does not give structural alignments, but if these are required they can be computed with tools like Dali after fast initial filtering with Progres. The impact of changing model hyperparameters is shown in Supplementary Table S1. The distribution of values across the embedding dimensions is shown in Supplementary Fig. S1.

In order to assess the accuracy of the model for structure searching, we follow a similar procedure to Foldseek (van Kempen *et al.* 2024). Since our model is trained on SCOPe domains, it is important not to use domains for training that appear in the test set. We select a random set of 400 domains from the Astral 2.08 40% sequence identity set for testing. No domains in the training set have a sequence identity of 30% or more to these 400 domains. This represents the realistic use case that the query structure has not been seen during training—e.g. it is a predicted or new experimental structure—but other domains in the family may have been seen during training. The easier case of searching with the exact domains used for training gives superior results that are not reported here, and the harder case of searching with completely unseen folds is discussed later.

**Figure 2.**
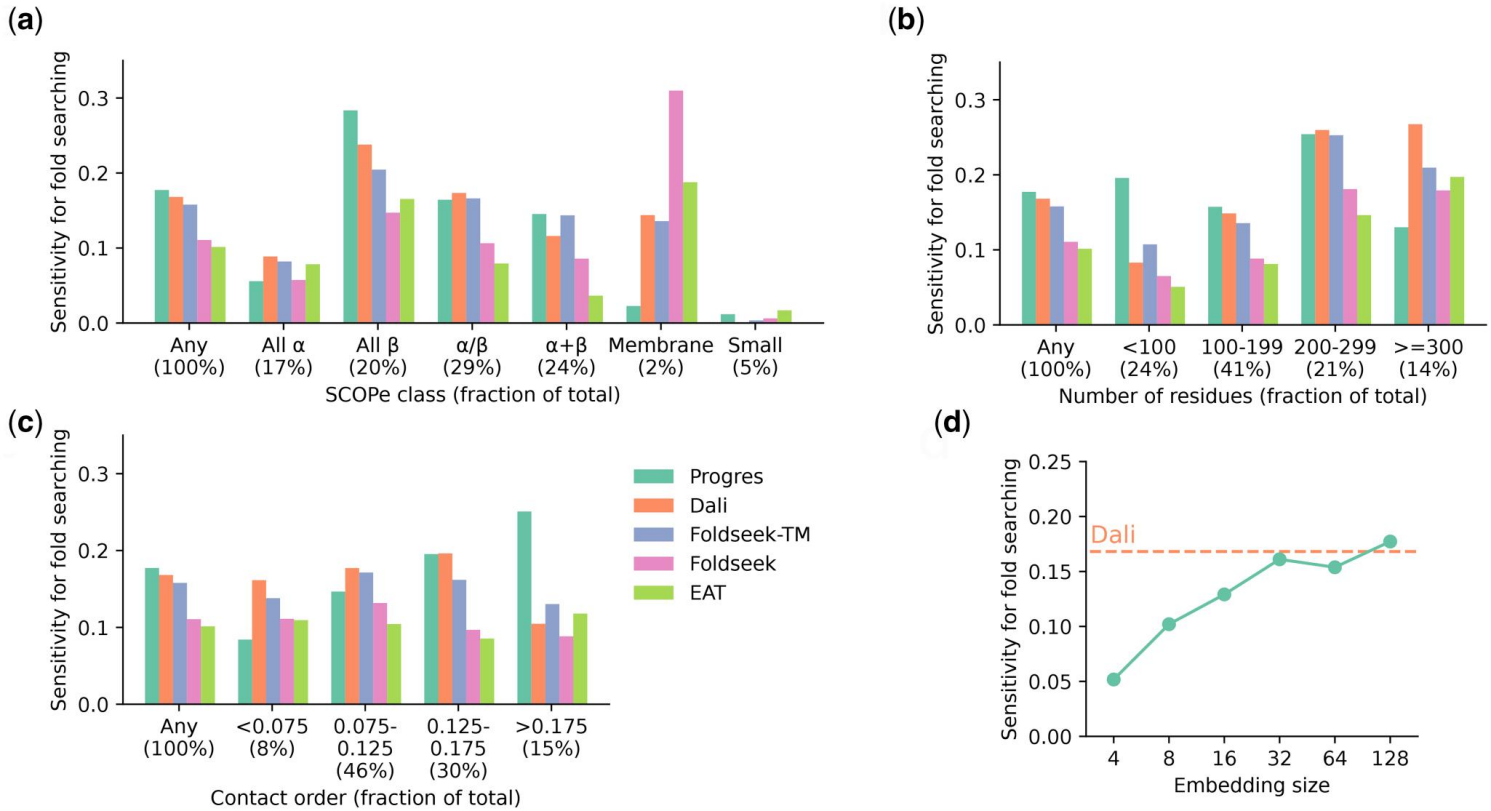
Model performance on different protein types. In each case the “Any” category is the same as in Table 1. (a) Sensitivity for fold searching by SCOPe class. The “Small” class contains proteins with little or no secondary structures. (b) Sensitivity for fold searching by protein sequence length. (c) Sensitivity for fold searching by contact order, a measure of the sequence separation of contacting residues. (d) Sensitivity for fold searching across different embedding sizes. A model was trained from scratch for each embedding size. See Supplementary Table S1 for further ablations.

As shown in Table 1, our model has sensitivity comparable to Dali (Holm 2020) and Foldseek-TM (van Kempen *et al.* 2024) for recovering domains in SCOPe from the same fold, superfamily, and family. Its strong performance at the fold-level indicates an ability to find remote homologs. Progres is more sensitive than the EAT (Heinzinger *et al.* 2022) and ESM-2 (Lin *et al.* 2023) protein language model embeddings, and also the baseline sequence searching method of MMseqs2 (Steinegger and Söding 2017). This indicates the benefits of comparing structures rather than just sequences for detecting homology. Figure 2a–c shows the performance across different SCOPe classes, protein sizes, and contact orders. Progres does particularly well on all-β domains, smaller domains and domains with higher contact order. This ability to do well in cases where residues separate in sequence form contacts is possibly due to the lack of primary sequence information in the embedding, compared to a method like Foldseek that retains the sequence order for searching. It has lower performance on membrane proteins and larger domains. As shown in Fig. 2d, performance drops when the number of embedding dimensions is below 32.

**Table 1.** Comparison of ability to retrieve homologous proteins from SCOPe.

Software	Searching sensitivity			Top 20 hits		Run time	
	Fold	Super-family	Family	Mean TM-align	Fraction correct folds	Single	All-v-all
Progres (this work)	0.177	0.706	0.877	0.621	0.853	1.3 s (CPU)	163 s (CPU), 127 s (GPU)
Dali (Holm 2020)	0.168	0.709	0.885	0.673	0.920	508 s	> 1 month
Foldseek-TM (van Kempen *et al.* 2024)	0.158	0.666	0.859	0.662	0.898	4.8 s	2 h 47 m
Foldseek (van Kempen *et al.* 2024)	0.111	0.644	0.850	0.656	0.889	2.3 s	250 s
TM-align fast (Zhang and Skolnick 2005)	0.100	0.594	0.806	0.688	0.847	390 s	∼23 days
3D-SURFER (Xiong *et al.* 2014)	0.046	0.140	0.349	0.511	0.560	7.2 s	24 h
EAT (Heinzinger *et al.* 2022)	0.101	0.615	0.843	0.627	0.825	34 s (GPU)	4 h 37 m (GPU)
ESM-2 (Lin *et al.* 2023)	0.014	0.221	0.477	0.546	0.598	28 s (GPU)	590 s (GPU)
MMseqs2 (Steinegger and Söding 2017)	0.001	0.165	0.433	0.488	0.390	0.9 s	17.1 s

A similar procedure to Foldseek (van Kempen *et al.* 2024) is followed for searching sensitivity with a set of 400 domains. For each domain, the fraction of true positives (TPs) detected up to the first incorrect fold is calculated (higher is better). TPs are same family in the case of family-level recognition, same superfamily and not same family in the case of superfamily-level recognition, and same fold and not same superfamily in the case of fold-level recognition. The mean of this fraction over all 400 domains is reported. Run time (single) is the time taken to search a structure of 150 residues (d1a6ja_ in PDB format) against all the 15 177 Astral 2.08 40% sequence identity set domains, with the database pre-prepared. Run time (all-v-all) is the time taken to calculate all pairwise distances between the 15 177 domains from structure. EAT, ESM-2, and MMseqs2 use sequence not structure for searching. 3D-SURFER and EAT are trained with structural information and may have seen proteins in the test set during training.

For searching a single structure against SCOPe on CPU the model is faster than Foldseek with most run time in Python module loading. For example, going from 1 to 100 query structures increases run time from 1.3 to 2.4 s. When searching with multiple structures, most run time is in generating the query structure embeddings. Consequently, the speed benefits of the method arise when searching a structure or structures against the pre-computed embeddings of a huge database such as the AlphaFold database (Aderinwale *et al.* 2022, Barrio-Hernandez *et al.* 2023, Durairaj *et al.* 2023, Procházka *et al.* 2024). The recent TED study split the whole AlphaFold database into domains using a consensus-based approach (Lau *et al.* 2024). We embed the TED domains clustered at 50% sequence identity and use FAISS (Douze *et al.* 2024) to considerably speed up the search time against the resultant database of 53 million structures. This allows a search time of a tenth of a second per query on CPU, after an initial data loading time of around a minute. Since we search exhaustively with FAISS, the results are not changed, though the approximate score calculation means the similarity score does vary slightly from the exact value. For the SCOPe test set used above, the mean difference between FAISS and exact similarity scores for the top hit is 0.006. As shown in Supplementary Fig. S2, the best TM-align score to the query among the top five hits has a mean of 0.80 across the SCOPe test set, with 94% being over 0.5. This indicates that searching is accurate even when using a large database.

**Figure 3.**
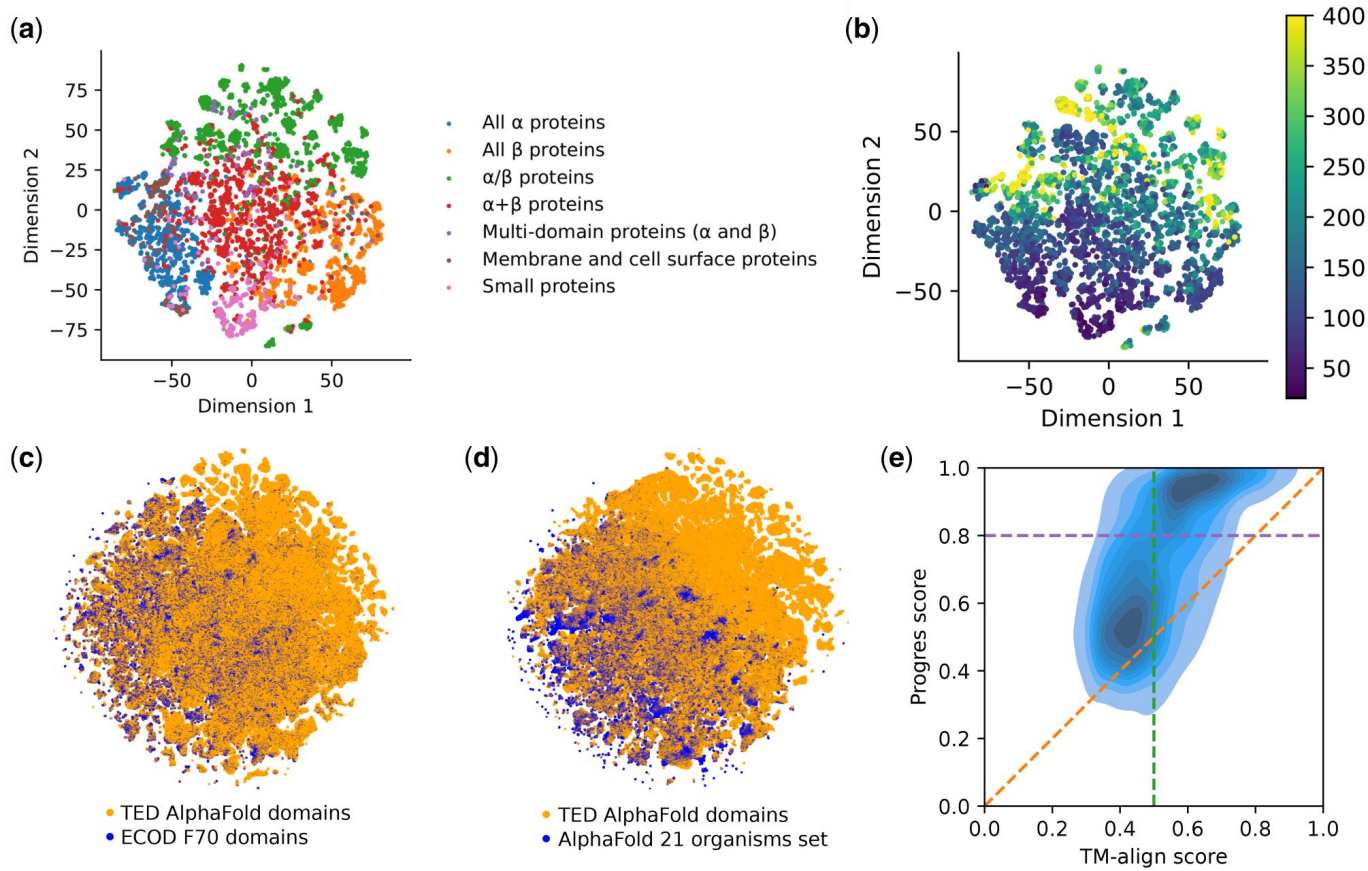
Exploring Progres embeddings. (a) 2D t-SNE embedding of the 128 dimensions of our model embedding for the Astral set of SCOPe domains clustered at 40% sequence identity (15 177 domains). This includes domains similar to both the training and test sets. The domains are coloured by SCOPe class. t-SNE was carried out using a perplexity value of 30. (b) The same data coloured by number of residues in the domain. The median length of domains is 149 residues. For colouring, the maximum number of residues in a domain is treated as 400. (c) 2D t-SNE of the AlphaFold database TED domains (Lau *et al.* 2024) clustered at 50% sequence identity and the ECOD F70 set of domains in the PDB (Cheng *et al.* 2014). 5 m (9%) of the TED domains are chosen randomly for the t-SNE for computational reasons. (d) A similar comparison of the TED domains to the AlphaFold 21 model organisms set (Varadi *et al.* 2022, Bordin *et al.* 2023). (e) Comparison of Progres score to TM-align score. For each of the 400 domains in the test set the top 200 matches in the Astral 40% sequence identity set according to TM-align are considered. The Pearson correlation coefficient is 0.60. The green line shows the TM-align score threshold of 0.5 indicating the same fold. The purple line shows the Progres score threshold of 0.8 indicating the same fold.

Figure 3 shows 2D t-SNE embeddings (van der Maaten and Hinton 2008) of the 128 dimensions of our model embedding. This shows the lower-dimensional protein fold space (Holm and Sander 1998, Kolodny *et al.* 2013, Nepomnyachiy *et al.* 2014) created by our embedding. SCOPe classes tend to cluster together, with α + β folds appearing between the all-α and all-β folds which show little overlap. There is a clear protein size gradient across the t-SNE embedding. A t-SNE embedding for the AlphaFold database TED domains compared to ECOD (Cheng *et al.* 2014) and the AlphaFold 21 organisms set (Varadi *et al.* 2022, Bordin *et al.* 2023) shows the volume of new structural information available in the AlphaFold database. The Progres score between two embeddings is the cosine similarity score normalized to run between 0 and 1, with 1 indicating identical embeddings. As shown in Fig. 3e, a Progres score of 0.8 indicates that two proteins share the same fold, analogous to a TM-align score of 0.5.

The web server provides an easy way to use Progres without installing software. The same options and databases are available as for the downloadable software. The results page provides visualization of query and hit domains, allows sorting and searching the results table for each domain, and gives download links for hit domains.

As an example of using the method, we consider a bacterial methyltransferase that modifies specific bases in the bacteria’s ribosome, granting resistance to streptomycin (PDB ID 3FRH) (Schmitt *et al.* 2009). This is shown in Fig. 1c. Since the 250 residue protein appears to consist of two domains, we use the Chainsaw option to split the domains and search them separately. When searching against CATH, the top hits are to domains from superfamily 3.40.50.150 for the larger C-terminal domain and 1.10.8.10 for the smaller N-terminal domain, matching the CATH classification for this structure. The larger domain also hits the Rossmann-like superfamily 3.40.50.720, which TM-align indicates as having the same fold, and the smaller domain hits a number of other helical bundles. Searching the larger domain against the AlphaFold TED domains gives 45 040 hits with Progres score at least 0.9, indicating a high likelihood of having the same fold. Of these, 1836 are not annotated in TED with the 3.40.50.150 superfamily and represent possible more distant relationships relevant to antibiotic resistance.

## 4 Discussion

The model presented here is trained and validated on protein domains; due to the domain-specific nature of the training it is not expected to work without modification on protein chains containing multiple domains, long disordered regions, or complexes. Fortunately, there are a number of tools such as Chainsaw (Wells *et al.* 2024), Merizo (Lau *et al.* 2023), and SWORD2 (Cretin *et al.* 2022) that can split query structures into domains. We integrate Chainsaw into Progres to allow automated splitting of query structures into domains, with each domain then searched separately. This can overcome issues that arise from searching with multiple domains at the same time, such as missing related proteins due to differing orientations of the domains. Splitting with Chainsaw takes a few seconds per query. As shown in Supplementary Fig. S3, the Progres embeddings are fairly robust to truncating residues from the termini, with truncations of 20 residues giving an embedding with a similarity of 0.8 to the full length domain embedding for 89% of domains with 200–299 residues. This means that minor inaccuracies in predicting domain boundaries are unlikely to cause a problem.

One issue with supervised learning on domains is whether performance drops when searching with domains that the model has not seen anything similar to during training. We trained an identical model on a different dataset where 200 domains were used for testing and domains were removed from the training set if they were from the same SCOPe superfamily as any of the testing domains. The fold, superfamily, and family sensitivities analogous to Table 1 are 0.190, 0.383, and 0.546, respectively. This indicates similar performance at finding distantly related folds, the main use of structure searching over sequence searching, though there is a drop in performance at finding closely-related domains. Hence, Progres is still useful in this context, e.g. for transferring function annotations from related folds. One challenge of training supervised methods on protein structure classifications is the difficulty of leaving enough data for training when holding out a completely distinct test set.

Aside from searching for similar structures, an accurate protein structure embedding has a number of uses. Fast protein comparison is useful for clustering large sets of structures, e.g. to identify novel folds in the AlphaFold database (Barrio-Hernandez *et al.* 2023, Durairaj *et al.* 2023, Lau *et al.* 2024). The embedding of a structure is just a set of numbers, and therefore can be targeted by differentiable approaches for applications like protein design. A decoder could be trained to generate structures from the embedding space (Ingraham *et al.* 2019, Guo *et al.* 2021), and a diffusion model to move through the embedding space. Properties of proteins such as evolution (Ding *et al.* 2019), topological classification (Taylor 2002), the completeness of protein fold space (Cossio *et al.* 2010), the continuity of fold space (Skolnick *et al.* 2009), function (Leman *et al.* 2023), and dynamics could also be explored in the context of the low-dimensional fold space. Structure embeddings could also be used to identify regions of unknown density in cryo-electron tomography studies. We believe that the extremely fast pairwise comparison allowed by structural embeddings is an effective way to take advantage of the opportunities provided by the million-structure era.

## Supplementary Material

vbaf042_Supplementary_Data

## Data Availability

A Python package allowing structure searching and generation of pre-embedded databases is available along with datasets, training scripts, and benchmarking scripts under a permissive license at https://github.com/greener-group/progres. The trained model and pre-embedded databases are available at https://zenodo.org/record/7782088. A web server for searching is available at https://progres.mrc-lmb.cam.ac.uk.
