# Supplementary material for: Fast protein structure searching using structure graph embeddings
Source: Bioinform Adv. 2024 Mar 5;5(1):vbaf042. doi: 10.1093/bioadv/vbaf042 (PMC11974391; doi:10.1093/bioadv/vbaf042)
Supplement: vbaf042_Supplementary_Data [file vbaf042_supplementary_data.pdf]

# Fast protein structure searching using structure graph embeddings

Joe G Greener and Kiarash Jamali

## Supplementary Data

| Ablation | Fold | Superfamily | Family |
|---|---|---|---|
| No ablation | 0.177 | 0.706 | 0.877 |
| 64 embedding dimensions | 0.154 | 0.673 | 0.864 |
| 32 embedding dimensions | 0.161 | 0.699 | 0.884 |
| 16 embedding dimensions | 0.129 | 0.654 | 0.850 |
| 8 embedding dimensions | 0.102 | 0.491 | 0.696 |
| 4 embedding dimensions | 0.052 | 0.181 | 0.353 |
| 12 layers | 0.150 | 0.709 | 0.902 |
| 4 layers | 0.148 | 0.645 | 0.841 |
| No $\tau$ angle node feature | 0.159 | 0.690 | 0.869 |
| No Gaussian noise | 0.072 | 0.516 | 0.738 |
| No sinusoidal positional encoding | 0.052 | 0.402 | 0.642 |
| Float32 database for searching | 0.177 | 0.706 | 0.877 |
| Superfamily holdout validation | 0.190 | 0.383 | 0.546 |

**Table S1** Model ablations. In each case a model is trained from scratch and the same testing procedure is used as in Table 1. No ablation is the model presented in the results with 128 embedding dimensions and 6 layers. The embedding dimension ablation is shown visually in Figure 2D. Removing the $\tau$ angle node feature means the network is E(3)-invariant not SE(3)-invariant. Superfamily holdout validation is described in the discussion.

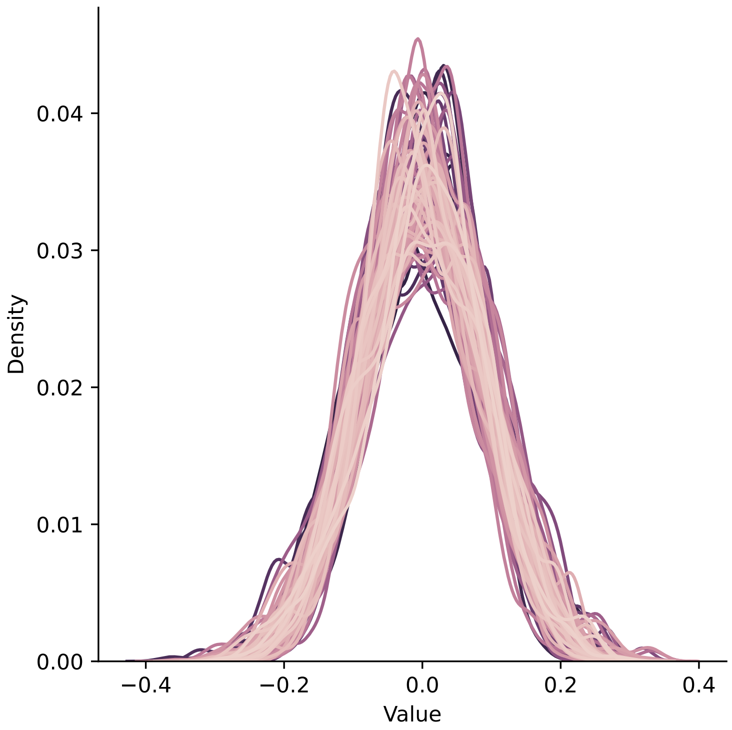


**Figure S1** Values of embedding dimensions. Each protein in the Astral 40% sequence identity set is embedded with Progres and the distribution of values in each of the 128 dimensions is shown.

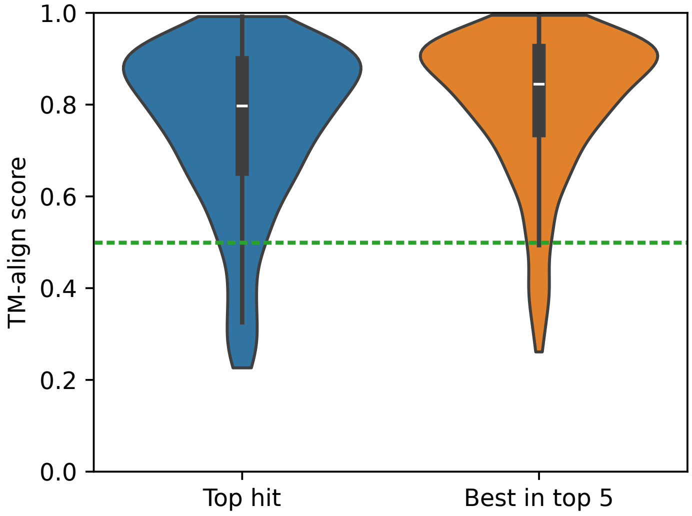


**Figure S2** Searching SCOPe domains against the AlphaFold database. Each of the 400 domains in the test set is searched against the AlphaFold database TED domains clustered at 50% sequence identity (53 million domains) and the TM-align score between the top hit and the query is calculated. This gives a mean TM-align score of 0.75 with 90% above 0.5. The highest TM-align score among the top 5 hits is also calculated and gives a mean of 0.80 with 94% above 0.5.

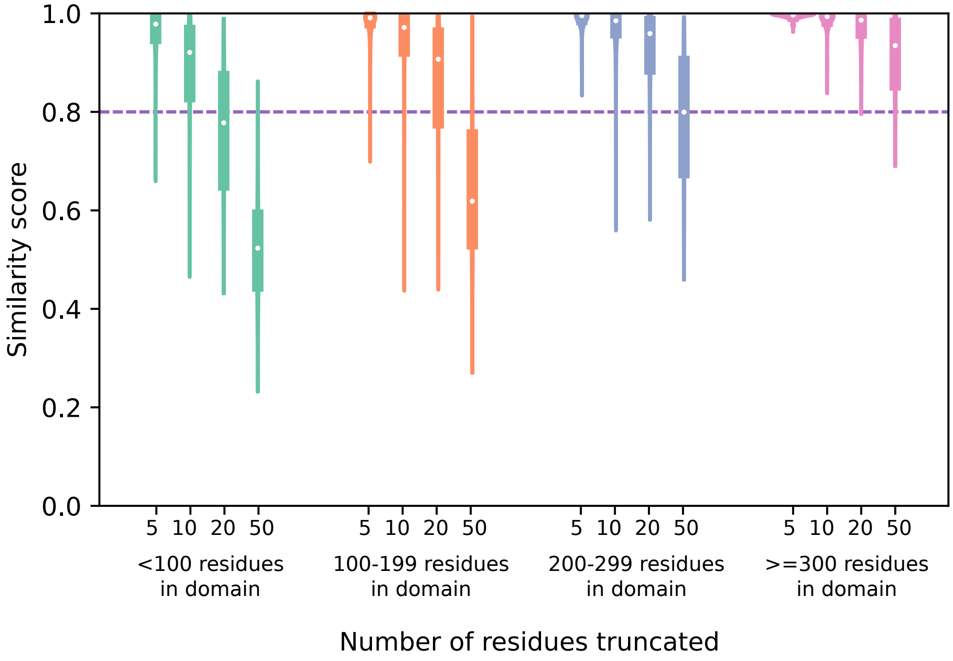


**Figure S3** The effect of truncating domains on Progres embeddings. For each of the 400 domains in the SCOPe test set a number of residues were removed from the N-terminus or the C-terminus and the truncated domain was embedded. The Progres similarity score to the full length domain was then computed. The results are categorised by the number of residues in the full length domain. The line shows a similarity score of 0.8, indicating the same fold (see Figure 3E).
